# Independent prognostic value of fascin immunoreactivity in stage I nonsmall cell lung cancer

**DOI:** 10.1038/sj.bjc.6600731

**Published:** 2003-02-18

**Authors:** G Pelosi, U Pastorino, F Pasini, P Maissoneuve, F Fraggetta, A lannucci, A Sonzogni, G De Manzoni, A Terzi, E Durante, E Bresaola, F Pezzella, G Viale

**Affiliations:** 1 Department of Pathology and Laboratory Medicine, University of Milan School of Medicine, Milan, Italy; 2 Department of Thoracic Surgery, European Institute of Oncology, Milan, Italy; 3 Department of Medical Oncology, University of Verona, Verona, Italy; 4 Department of Statistics and Epidemiology, European Institute of Oncology, Milan, Italy; 5 Department of Pathology, ‘Cannizzaro Hospital’, Catania, Italy; 6 Department of Pathology, City Hospital, Verona, Italy; 7 Department of General Surgery, University of Verona, Verona, Italy; 8 Department of Thoracic Surgery, City Hospital, Verona, Italy; 9 Nuffield Department of Clinical Laboratory Science, John Radcliffe Hospital, Headington, Oxford, UK

**Keywords:** fascin, nonsmall cell lung cancer, immunocytochemistry, prognosis, metastasis, angiogenesis

## Abstract

Fascin-1, the most expressed form of fascin in vertebrate tissues, is an actin-bundling protein that induces cell membrane protrusions and increases motility of normal and transformed epithelial cells. Very few data are available on the role of this protein in nonsmall cell lung cancer (NSCLC). Two hundred and twenty patients with stage I NSCLC and long-term follow-up were evaluated immunocytochemically for fascin expression. Overall, variable fascin immunoreactivity was detected in 98% of 116 squamous cell carcinomas, in 78% of 96 adenocarcinomas, in 83% of six large cell carcinomas, and in the two adenosquamous carcinomas under study. Neoplastic emboli were commonly decorated by the antifascin antibody (*P*<0.001), also when the surrounding invasive carcinoma was unreactive. Fascin immunoreactivity correlated with high tumour grade (*P*=0.017) and, in adenocarcinomas, with high Ki-67 labelling index (*P*=0.021). Adenocarcinomas with a prevalent bronchiolo-alveolar *in situ* component were less commonly immunoreactive for fascin than invasive tumours (*P*=0.005). Contralateral thoracic or distant metastases were associated significantly with diffuse (>60% immunoreactive tumour cells) fascin expression in adenocarcinomas (*P*=0.043), and marginally with strong fascin immunostaining in squamous cell carcinomas (*P*=0.13). No associations were noted with any other clinicopathological variables tested. Patients with tumours showing diffuse (>60% immunoreactive neoplastic cells) and/or strong immunoreactivity for fascin had a shorter survival (*P*=0.006 for adenocarcinomas and *P*=0.026 for squamous cell carcinomas), even after multivariate analysis (*P*=0.014 and 0.050, respectively). The current study documents for the first time that fascin is upregulated in invasive and more aggressive NSCLC, being an independent prognostic predictor of unfavourable clinical course of the disease. Targetting the fascin pathway could be a novel therapeutic strategy of NSCLC.

Lung cancer is one of the leading causes of morbidity and death worldwide. Patients with pathological stage I nonsmall cell lung cancer (NSCLC), however, have the best life expectation, because they lack major adverse prognostic factors, as incomplete surgical resection and lymph node or distant metastases. The current standard treatment for these patients is complete removal of the primary tumour without any additional adjuvant therapy. The long-term survival rate, however, is only from 55 to 72%, mainly because of the occurrence of local relapses and distant metastases (Nesbitt *et al*, 1995; Mountain, 1997; D'Amico *et al*, 1999).

Interaction among cancer cells, extracellular matrix proteins, and endothelial cells plays a fundamental role in the development of local invasion and distant metastases (Aznavoorian *et al*, 1993; Alessandro *et al*, 1996; Liotta and Kohn, 2001). Invasive tumour cells are often characterised by changes in cell shape with the appearance of membrane protrusions, loss of anchorage dependency, and loss of cell–cell adhesion and junctional communications. Many of these changes are because of rearrangements of the cytoskeletal microfilaments due to the action of several types of actin cross-linking proteins (Maekawa *et al*, 1988; Tilney *et al*, 1998).

Among these molecules, fascins (reviewed by Kureishy *et al*, 2002) are implied in the organisation of two major forms of actin-based structures that include either cortical cell protrusions, such as filopodia, spikes, lamellipodial ribs, dendrites and microvilli, or cytoplasmic microfilament bundles (Kureishy *et al*, 2002). The former structures are thought to play an active role in cell–matrix adhesion, cell interactions and cell migration, whereas the latter participate in maintaining cell architecture (Kureishy *et al*, 2002). In humans, three different forms of fascin are currently known, referred to as the vertebral fascin or fascin-1, the retinal fascin or fascin-2 (with 56% of homology to fascin-1), and the testis fascin as fascin-3 (with 27% homology to fascin-1). While fascin-1 is distributed in most vertebrate tissues, fascin-2 is circumscribed to the retina (Tubb *et al*, 2000; Wada *et al*, 2001), and fascin-3 is expressed specifically in the testis (Tubb *et al*, 2002). The human fascin-1 gene (FSCN1), located at chromosome 7q22 (Duh *et al*, 1994), encodes for an evolutionarily highly conserved 55-kDa actin-bundling protein (Duh *et al*, 1994; Mosialos *et al*, 1994; Yamashiro-Matsumura and Matsumura, 1985) that induces membrane protrusions at the leading edges of the cells and greatly increases cell motility (Tao *et al*, 1996; Adams, 1997; Yamashiro *et al*, 1998). This protein is normally expressed by cells characterised by different types of membrane protrusions, such as neurons, glial cells and dendritic cells (Mosialos *et al*, 1994; Ross *et al*, 1998, Al-Alwan *et al*, 2001; Cohan *et al*, 2001), or by actively migrating cells, such as endothelial cells of the microvessels, which are extensile cells capable of migrating during angiogenesis (Mosialos *et al*, 1994; Tao *et al*, 1996, Pinkus *et al*, 1997; Hu *et al*, 2000). In normal epithelial cells, the fascin-1 expression level (later on indicated as fascin only) is usually low, but is often upregulated in several types of human neoplasms, such as breast (Sun *et al*, 1997; Grothey *et al*, 2000a, b; Guvakova *et al*, 2002), ovary (Hu *et al*, 2000) and pancreas carcinomas (lacobuzio-Donahue *et al*, 2002; Maitra *et al*, 2002), and skin tumours (Goncharuk *et al*, 2002). Very few data, however, are available on fascin expression in NSCLC. Recently, Del Rosario *et al* (2001) have reported in abstract form that fascin immunoreactivity correlates with tumour grade, but not tumour stage, death rate and survival of NSCLC patients. In the present study, we first investigated fascin immunoreactivity in NSCLC, comparing two different commercially available monoclonal antibodies in a retrospective series of 220 chemotherapy-naive patients with stage I disease. We document that fascin is overexpressed by most NSCLC, correlates with a tumour invasive phenotype, and is an independent prognostic indicator of reduced survival.

## MATERIALS AND METHODS

### Patients

The study population includes 220 unselected consecutive patients (199 males and 21 females) with stage I NSCLC treated at the Department of Thoracic Surgery of the ‘Ospedale Civile Maggiore’ in Verona (Italy) between 1987 and 1993. For each case, all paraffin blocks were retrieved and original haematoxylin and eosin-stained sections reviewed. Complete clinical follow-up for survival analysis was available for all patients, with a mean duration of 75.7±44.4 months (median 80 months; range 2–159 months). Recurrent disease was observed in 109 (49.5%) patients and 91 (41.4%) died of the disease.

Inclusion criteria for entry into the study included p-stage IA (95 men and 11 women) and IB (104 men and 10 women) according to the revised international system for staging lung cancer (Mountain, 1997), radical surgery, no (neo)-adjuvant therapy, a minimum 30-day postoperative survival, and a minimum follow-up of 5 years.

The patients’ age ranged from 35 to 80 years for men (mean±s.d.: 62.5±8.3 years; median: 63 years) and from 47 to 76 years for women (mean±s.d.: 61.3±7.3 years; median: 62 years). According to previously refined pathological criteria for lung tumours (Travis *et al*, 1999), there were 116 (52.7%) squamous cell carcinomas, 96 (43.6%) adenocarcinomas, six (2.8%) large cell carcinomas, and two (0.9%) adenosquamous carcinomas. Subtyping of adenocarcinomas was performed according to the WHO and Shimosato's classifications (Shimosato, 1994; Travis *et al*, 1999). Eleven (5.0%) carcinomas were well differentiated (10 adenocarcinomas and one squamous cell), 121 (55.0%) moderately differentiated (76 squamous cell, 43 adenocarcinomas, and two adenosquamous), and 88 (40.0%) poorly differentiated (43 adenocarcinomas, 39 squamous cell, and six large cell carcinomas). In homolateral thoracic recurrences there was a prevalence (17 out of 23) of adenocarcinomas, and in controlateral thoracic recurrences a prevalence (19 out of 27) of squamous cell carcinomas, whereas distant metastases were almost equally distributed between the two tumour types (24 out of 52 *vs* 28 out of 52) respectively: χ^2^=9.908, *P*=0.007). Complete information about the smoking history of the patients was not available at the time of the present study.

### Immunocytochemistry and evaluation of data

Formalin-fixed and paraffin-embedded tissue samples obtained at surgery were investigated. Tumours up to 2 cm in size were entirely embedded and immunostained; at least two representative tissue blocks were investigated in larger neoplasms. Samples of normal pulmonary parenchyma from patients with unrelated, nonmalignant lung diseases, and peritumoural lung tissue from the same cohort of NSCLC patients provided the control groups for noncarcinoma and carcinoma patients, respectively.

Fascin was immunolocalised on 4 *μ*m-thin tissue sections using two monoclonal antibodies, clone IM20 (Novocastra Laboratories, Newcastle upon Tyne, UK) that recognises the C-terminal region of the molecule, and clone 55k-2 (Dako, Glostrup, Denmark) that reacts with a 55-kDa protein in Western blots of HeLa, normal rat kidney and gerbil fibroma cell lysates (Yamashiro-Matsumura and Matsumura, 1986). The latter antifascin antibody was also found to immunoblot bacterially expressed human homolog singed gene (*hsn*) product and a 55-kDa protein from cell lysates of peripheral blood dendritic cells (Duh *et al*, 1994; Mosialos *et al*, 1996). The specificity of the clone IM20 has been evaluated on human tonsil for Western blot analysis, with a molecular weight detected at approximately 55-kDa (Novocastra Laboratories, personal communication, 2002, Figure 1

**Figure 1:**
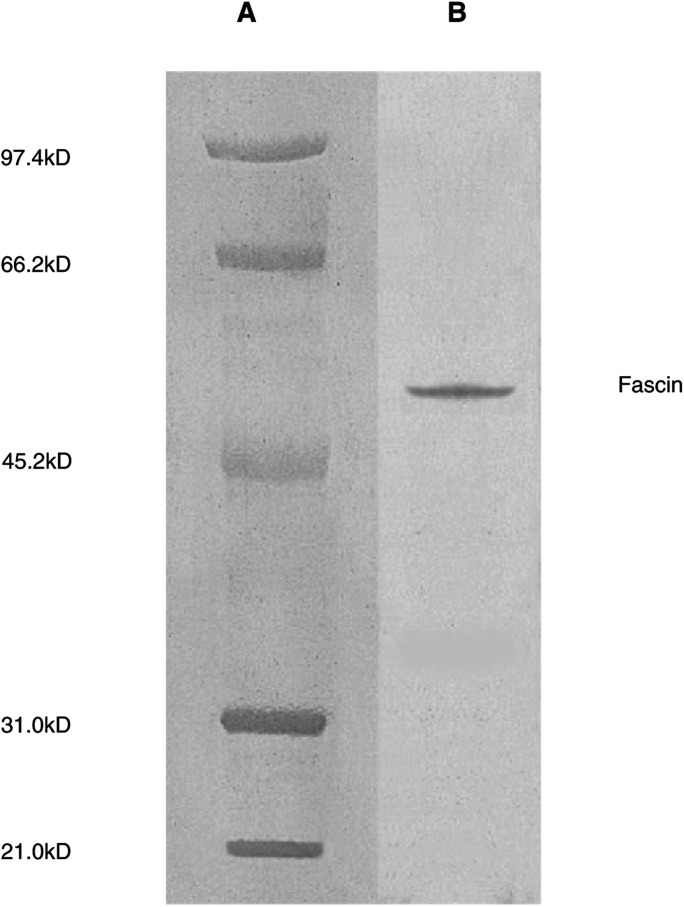
Western blot analysis of human tonsil using NCL-fascin (clone IM20). Lane **A**, molecular weight markers. Lane **B**, tonsil immunoblotted with NCL-FASCIN antibody. This image was supplied by courtesy of Novocastra Laboratories Ltd, Newcastle upon Tyne, UK.

). After blockage of endogenous peroxidase activity with 5% hydrogen peroxide and heat-mediated antigen retrieval with 1mM EDTA pH 8 (Sigma Chemical Co., St Louis, MO, USA) in a microwave oven at 750 W for 12 min, the sections were reacted overnight at 4°C with the primary antibodies at a dilution of 2.5 *μ*g ml^−1^ in Tris-buffered saline. Detection steps were performed using a commercially available kit (Dako EnVision Plus-HRP, Dako), according to the manufacturer's instructions. Peroxidase activity was developed with 3,3′-diaminobenzidine-copper sulphate (Sigma Chemical Co., St Louis, MO, USA) to obtain a brown–black end product. Tumour proliferative fraction was assessed by Ki-67 immunostaining and the percentage of reactive cells was recorded as the labelling index (Pelosi *et al*, 1996).

To colocalise fascin and Ki-67 antigen on the same tumour samples, representative blocks of NSCLC were double stained for fascin using the 55k-2 clone and for Ki-67 antigen. After overnight reaction with fascin and development with the Dako EnVision Plus-HRP kit and 3-amino-ethylcarbazole (Sigma) as a chromogen to yield a red end product, tissue sections were reacted with Ki-67 antigen (clone Mib-1, Immunotech, Marseille, France, 10 *μ*g ml^−1^) for 1 h at room temperature. Detection was performed using the Dako EnVision Plus-HRP kit and 3,3′-diaminobenzidine-copper sulphate to obtain a brown–black end product. This labelling procedure was chosen because nuclear antigens, such as Ki-67, are best highlighted in double-imunostaining experiments if developed in a more brilliant black–brown product, whereas cytoplasmic antigens may be easily evidenced also using a lighter red development. Microwave oven heating at 750 W for 5 min was used to block the residual enzyme activity after the first development reaction (Lan *et al*, 1995). Vascular invasion in NSCLC was highlighted by CD34 immunostaining of endothelial cells using standard immunohistochemical methods. The specificity of all immunoreactions was double-checked by substituting the primary antibodies with nonrelated isotypic mouse immunoglobulins at a comparable dilution, and with normal serum alone (Burry, 2000). Immunoreactivity of macrophages, dendritic cells of the bronchus-associated lymphoid tissue, and endothelial cells of microvessels was used as internal positive controls for fascin.

Two observers (GP and AS) evaluated fascin immunoreactivity of tumour cells independently and blindly, without knowledge of the patients’ identity or clinical outcome. Results were expressed semiquantitatively: as negative to low immunoreactivity, if staining was either completely absent or observed in less than 5% of cells; as moderate immunoreactivity if staining was observed in 5–60% of tumour cells; and as high immunoreactivity if staining was recognised in more than 60% of tumour cells. Also, the intensity of fascin immunostaining was simulataneously graded as low if a faint to distinct granular labelling was detectable throughout the cytoplasm, but less intense than seen in the normal internal controls (endothelial and dendritic cells), or strong if it was of the same or greater intensity than the latter.

### Statistical analysis

Associations of categorical variables were evaluated by Fisher's exact *t*-test or *χ*^2^ test (applying Yates’ correction for continuity). All correlation tests were performed using Spearman's rank test (*r*). Intra- and interobserver reproducibility between two independent observers was assessed as previously reported in detail (Pelosi *et al*, 1996). Differences in immunostaining between the two antibodies in the whole series of NSCLC were evaluated with Wilcoxon's signed-rank test for pairs. Survival estimates were calculated with Kaplan–Meier's method and compared by Cox–Mantel's log rank test. The comparative importance of explanatory variables on survival time was evaluated by means of Cox's proportional hazard regression model. The following variables were examined for overall (OS) and disease-free survival (DFS) analysis: age, sex, performance status, clinical symptoms, tumour grade, tumour size, pathological stage, proliferative fraction, and vascular invasion. All the analyses were performed using the SAS statistical software (SAS Institute, Inc., Cary, NC, USA). All *P*-values were based on two-sided testing.

## RESULTS

**Figure 2:**
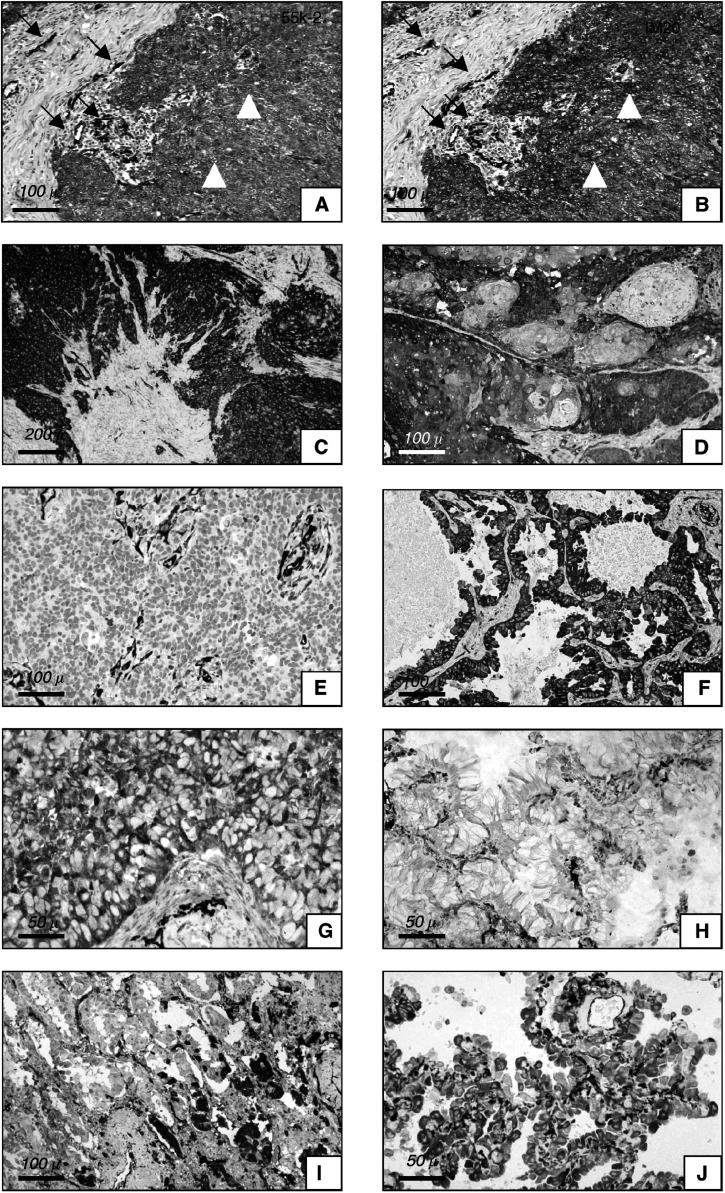
(**A–J**) Comparison of immunostaining between monoclonal antibodies 55k-2 from Dako (**A**) and IM20 from Novocastra (**B**) in the same histological field of NSCLC: both tumour cells (white triangles) and normal endothelial cells (arrows) are similarly highlighted by the two reagents with no significant differences. Squamous cell carcinomas usually show diffuse immunostaining for fascin (**C**), whereas horn pearls are less immunoreactive (**D**). Only rarely squamous cell carcinomas lack fascin expression: in these cases, immunoreactivity of endothelial cells serves as internal positive control (**E**). (Panels **C** and **D** represent different areas of the same squamous cell carcinoma according to the different differentiation grade, whereas panel **E** refers to another case of squamous cell carcinoma showing very poor differentiation). In adenocarcinomas, invasive patterns of papillary (**F**) and solid (**G**) types show commonly immunolabelling for fascin, whereas the bronchiolo-alveolar pattern, either nonmucinous or mucinous (**H**), is generally unreactive for fascin even when this component is present in mixed forms along with an invasive pattern of acinar type (**I**). Nonmucinous type, however, may show occasional immunoreactivity for fascin (**J**). (Panels **F–J** belong to different cases of adenocarcinoma). (**A**: 55k-2 monoclonal antibody; **B--J**: IM20 monoclonal antibody. All immunostains were performed with diaminobenzidine, and then counterstained with haematoxylin. Scale bars are reported in the bottom on the left of every panel.)

Fascin immunoreactivity appeared as a fine, granular to diffuse cytoplasmic staining, in both normal and neoplastic cells. IM20 and 55k-2 monoclonal antibodies did not differ significantly with regard to both the number of immunoreactive cells as evaluated semiquantitatively using three scoring categories (*P*=0.75) and the immunostaining intensity as evaluated in two subjective categories by comparison with internal positive controls (*P*=0.31) (Figure 2 A, B

). Accordingly, very high correlation was noted between the two antibodies for both evaluation systems of immunoreactivity (*r*=0.95 for the number of immunoreactive cells, *P*<0.001; *r*=0.91 for the immunostaining intensity, *P*<0.001).

In the normal lung, fascin immunoreactivity was invariably found in endothelial cells of the microvessels in the bronchial and alveolar walls, in dendritic cells of the mucosa-associated lymphoid tissue, and in tissue macrophages. Endothelial cells of major pulmonary vessels and bronchial and alveolar epithelia were consistently unreactive, although the basolateral membranes of bronchial epithelial cells were occasionally decorated by the antiserum. No differences in fascin immunoreactivity were seen in the normal pulmonary parenchyma from patients with unrelated, nonmalignant lung diseases, and in the peritumoural lung tissue from the same cohort of NSCLC patients.

Overall, fascin immunoreactivity was detected in 196 out of 220 (89%) NSCLC, including 75 out of 96 (78%) adenocarcinomas, 114 out of 116 (98%) squamous cell carcinomas, five out of 6 (83%) large cell carcinomas, and the two adenosquamous carcinomas investigated (*χ*^2^=22.4, *P*<0.001). Independent of tumour type, fascin-immunoreactive tumours showed heterogeneity both in the percentage of labelled cells and in the intensity of immunostaining (Table 1

**Table 1:** Distribution of fascin immunoreactivity in the 220 NSCLC

	**Immunoreactive cells (%)**^a^			**Immunolabelling intensity (%)**^b^		
**Histotype**	**None**	**5–60%**	**>60%**	**None**	**Low**	**High**
Adenocarcinoma (*N*=96)	21 (22)	41 (43)	34 (35)	21 (22)	43 (45)	32 (33)
Adenosquamous (*N*=2)	0 (0)	0 (0)	2 (100)	0 (0)	0 (0)	2 (100)
Squamous cell carcinoma (*N*=116)	2 (2)	14 (12)	100 (86)	2 (2)	45 (47)	69 (72)
Large cell carcinoma (*N*=6)	1 (17)	1 (17)	4 (66)	1 (17)	0	5 (83)

a *χ*^2^=61.8, *P*<0.001.

b *χ*^2^=34.7, *P*<0.001.

and Figure 2 C–J). Immunoreactivity was sometimes markedly enhanced at the tumour–host interface, whereas horn pearls of squamous cell carcinomas were less immunoreactive than adjacent neoplastic nests. A close correlation was found between the percentage of fascin-immunoreactive cells and the staining intensity within individual tumours (*r*=0.79, *P*<0.001). Evaluation of intraobserver reproducibility did not show significant differences among the different tumour types, and a high interobserver correlation was noted in both scoring fascin immunoreactive cells and grading the intensity of immunostaining (*r*=0.96, *P*<0.001).

Adenocarcinomas with prevalent (⩾70%) bronchiolo-alveolar component were less commonly (two out of seven cases) immunoreactive for fascin (Figure 2, H, I) than those with prevalent invasive components of acinar (18 out of 23) papillary (24 out of 31), or solid (28 out of 31) types (*χ*^2^=19.44, *P*=0.005) (Figure 2, F, G). The bronchiolo-alveolar *in situ* component at the periphery of adenocarcinomas also was less immunoreactive for fascin than the invasive components of acinar, papillary, or solid types (Figure 2I). Reduced fascin imunoreactivity was more commonly encountered in the case of nonmucinous (16 out of 22 cases) than in mucinous (one out of 5 cases) bronchiolo-alveolar patterns (*P*=0.047), even though the former could show occasional fascin immunoreactivity (Figure 2J).

**Figure 3:**
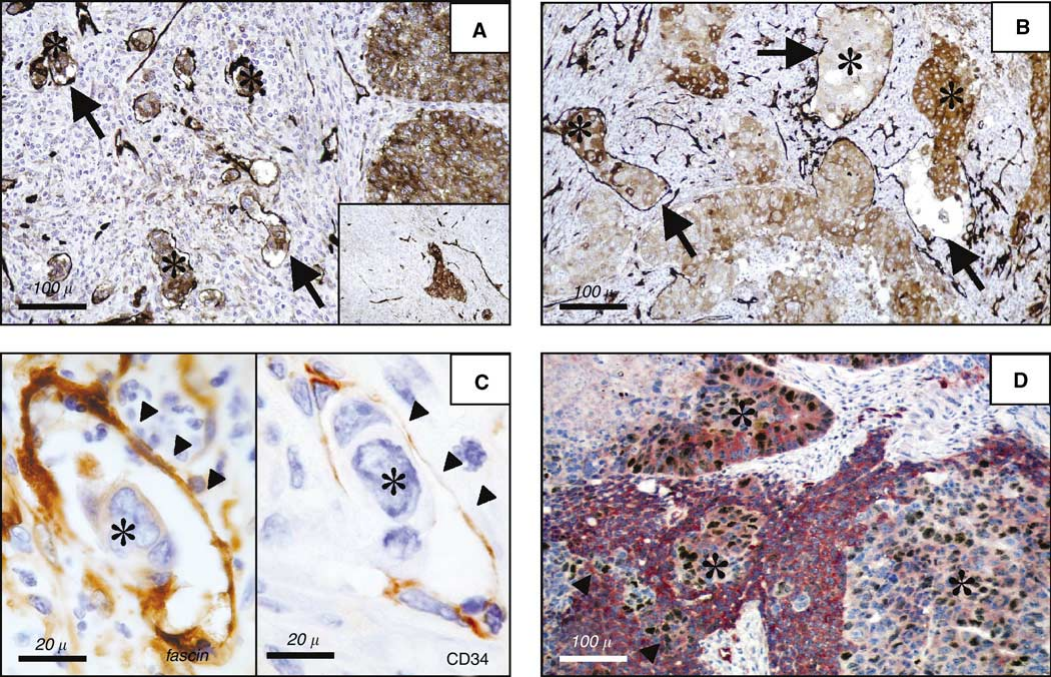
(**A–D**) Neoplastic emboli (asterisks) within vascular channels (arrows) of both squamous cell carcinoma (two tumour nests infiltrating the stroma are also present on the right of the Figure) (**A**) and adenocarcinomas (**B**). Tumour emboli are strongly immunoreactive for fascin, even when the remainder tumour is negative for the marker (**A**, inset). In a case of adenocarcinoma, the same sinusoidal space, engulfed by tumour cells (asterisks), is delimited by CD34-positive endothelial cells (black triangles) and strongly reacts for fascin (**C**). Double immunostaining for fascin (red) and Ki-67 antigen (black–brown) shows that highly proliferating tumour cell foci (asterisks) are downregulating fascin expression, whereas adjacent neoplastic nests show either lower proliferative activity (black triangles) or strong immunoreactivity for fascin (**D**). (**A–D**: IM20 monoclonal antibody; in **D**, also Mib-1 monoclonal antibody. Immunoperoxidase staining was performed with diaminobenzidine to obtain a black–brown end product (**A–D**) or amino-ethylcarbazole to obtain a red end product (**D**), and then counterstained with haematoxylin. Scale bars are reported in the bottom on the left of every panel.)

Sixty-five tumours (34 adenocarcinomas and 31 squamous cell carcinomas) showed vascular invasion, highlighted by CD34 immunostaining of endothelial cells. The neoplastic emboli were intensely labelled by the anti-fascin antibody (Figure 3 A, B

), independent of the staining intensity of the surrounding tumour cells (Figure 3A, inset), and were often conatined in CD34-immunoreative sinusoidal spaces (Figure 3C). Of these 65 tumours, 57 (26 adenocarcinomas and 31 squamous cell carcinomas) showed immunostaining of both the neoplastic emboli and of the invasive neoplastic nests, whereas eight out of 65 tumours (all adenocarcinomas) did not show any fascin immunoreactivity in either component (*P*<0.001). Contralateral thoracic or distant metastases (32 out of 79 in adenocarcinomas and 47 out of 79 in squamous cell carcinomas, *P*=0.23) were associated significantly with diffuse (>60% immunoreactive tumour cells) fascin expression in adenocarcinomas (*P*=0.043), and marginally with strong fascin immunostaining in squamous cell carcinomas (*P*=0.13).

Well-differentiated NSCLC considered as a whole were more commonly (five out of eight cases) fascin-negative, whereas poorly differentiated tumours more often (46 out of 57 cases) exhibited diffuse fascin immunoreactivity in more than 60% neoplastic cells (*P*=0.017). In adenocarcinomas, the percentage of fascin-immuno-reactive cells correlated significantly with the Ki-67 labelling index (*P*=0.021), whereas this correlation was weaker in squamous cell carcinomas (*P*=0.14). When stratified for fascin levels of immunostaining, adenocarcinomas with high (>60%) fascin immunoreactivity exhibited more pronounced proliferative activity (mean±s.d.: 27.9±16.5%; median: 27%) than tumours with negative to low (up to 30%) fascin immunoreactivity (mean±s.d. 20.4±13.4%; median: 20%) (*P*=0.028). This association, however, evidenced a wide dispersion of the values, as shown by their low correlation coefficient (*r*=0.23, d.f.=94). As a matter of fact, double-immunostaining experiments indicated that the number of fascin-positive cells was usually by far higher than Ki67-positive cells within individual tumours, whereas nests of actively proliferating tumour cells showed a consistent decrease of fascin immunoreactivity (Figure 3D). No other associations with gender, age, clinical symptoms, and performance status of the patients, or with tumour diameter, pathological stage, and cell subtyping were observed.

**Figure 4:**
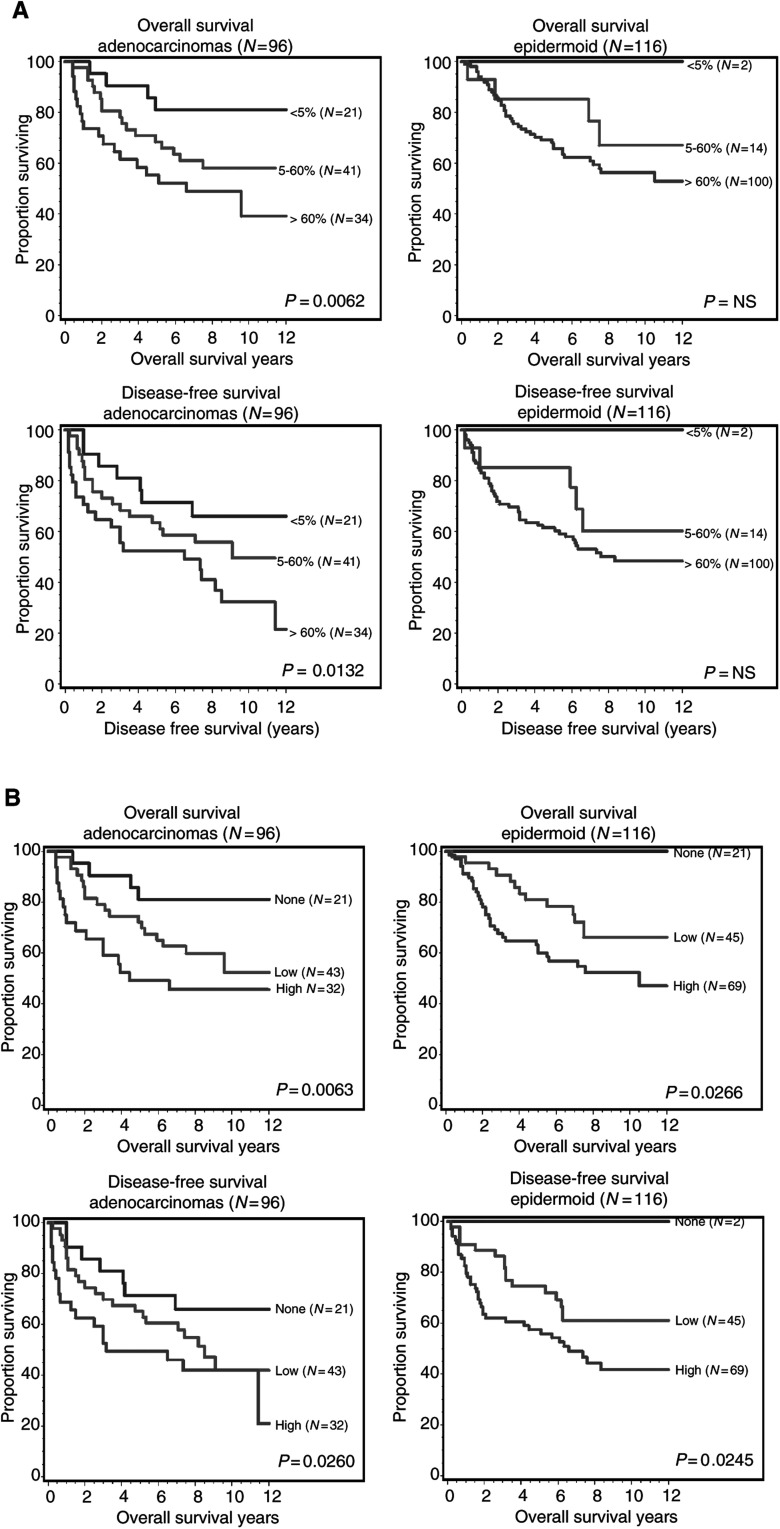
(**A**,**B**) Overall and disease-free survival curves according to the number of fascin-immunoreactive tumour cells (**A**) and immunostaining intensity (**B**) in both adenocarcinomas and epidermoid or squamous cell carcinomas.

Patients with tumours showing diffuse (i.e. in more than 60% neoplastic cells) or strong immunoreactivity for fascin had shorter OS (*P*=0.006 for adenocarcinomas and *P*=0.026 for squamous cell carcinomas) and DFS (*P*=0.013 and 0.024, respectively) (Figure 4 A, B

). Other prognostic factors were tumour stage and diameter, performance status, and clinical symptoms (Table 2

**Table 2:** Univariate prognostic value of tumour variables for survival

		**Adenocarcinoma**		**Squamous cell carcinoma**	
**Variables**	**Categories**	**OS**	**DFS**	**OS**	**DFS**
Sex	M/F	0.1993	0.0632	0.4113	0.7377
Age	Continuous	0.8378	0.1045	0.8748	0.2793
Tumour grade	G1/G2/G3	0.6593	0.3657	0.2807	0.1915
Tumour stage	T1a/T1b	0.0466	0.1754	0.4964	0.1248
Tumour diameter	Continuous	0.0236	0.0565	0.0752	0.0188
Performance status	Continuous	<0.0001	0.0005	0.5026	0.5629
Symptoms	No/local/general	0.0002	0.0004	0.2854	0.3134
Ki-67	Continuous	0.2653	0.4655	0.2551	0.0910
Vascular invasion	Present *vs* absent	0.8723	0.9899	0.0202	0.0156
Fascin-reactive cells	None/5*–*60%/>60%	0.0062	0.0132	0.2367	0.1852
	<5% *vs* >60%	0.0129	0.0219	–	–
Fascin intensity	None/low/high	0.0063	0.0260	0.0266	0.0245

Fascin intensity: None, no immunoreactivity; low, weak to moderate immunoreactivity; high: intense immunoreactivity.

). Multivariate analyses showed that diffuse fascin immunoreactivity, performance status, and clinical symptoms were independent prognostic factors for both OS and DFS in patients with adenocarcinomas. Conversely, strong fascin immunostaining and vascular invasion emerged as independent prognostic factors in patients with squamous cell carcinomas (Table 3

**Table 3:** Multivariate analysis

**Variables**	**Categories**	**HR**^a^	**95% CI**^a^	***P* value**
*Adenocarcinomas*				
Age	Continuous	0.97	0.94–1.01	0.0957
Sex	F *vs* M	0.39	0.13–1.17	0.0916
Fascin-reactive cells	<5% *vs* >60%	4.48	1.35–14.9	0.0144
Tumour grade	G1/G2/G3	0.74	0.40–1.36	0.3303
Tumour stage	T1B *vs* T1A	1.82	0.42–7.83	0.4213
Tumour diameter	Continuous	0.79	0.17–3.73	0.7649
Performance status	Continuous	0.94	0.89–0.99	0.0233
Symptoms	Present *vs* absent	2.96	1.07–8.22	0.0368
Vascular invasion	Present *vs* absent	1.23	0.59–2.57	0.5901
Ki-67 index	Continuous	1.01	0.99–1.03	0.1553
				
*Squamous cell carcinomas*				
Age	Continuous	1.02	0.98–1.06	0.3874
Sex	F *vs* M	0.79	0.18–3.37	0.7450
Fascina intensity	High *vs* low	1.83	1.00–3.35	0.0516
Tumour grade	G1/G2/G3	1.02	0.57–1.83	0.9518
Tumour stage	T1B *vs* T1A	0.98	0.37–2.56	0.9612
Tumour diameter	Continuous	1.11	0.88–1.40	0.3914
Performance status	Continuous	1.01	0.97–1.04	0.6433
Symptoms	Present *vs* absent	1.42	0.68–2.96	0.3562
Vascular invasion	Present *vs* absent	1.19	1.01–3.62	0.0481

a Hazards ratio (HR) and 95% confidence intervals (CI) were obtained from a Cox proportional regression model.

).

## DISCUSSION

The current investigation documents that fascin is upregulated in most (89%) NSCLC, and correlates with higher tumour grade and higher proliferative fraction. Diffuse or intense fascin immuno-reactivities are independent unfavourable prognostic factors in patients with adenocarcinoma and squamous cell carcinomas, respectively. Finally, our data suggest that fascin is likely involved in tumour invasiveness and vascular invasion.

This is, to our knowledge, the first study in which the immunoreactivities of two different, commercially available monoclonal antibodies against fascin are evaluated on the same large series of human carcinomas. In our study, the IM20 clone gave the same brilliant decoration of targeted cells as 55k-2 (Figure 2A, B), and the final results within individual tumours did not change significantly between the two reagents in terms of both number of immunoreactive cells as evaluated semiquantitatively in three categories (negative to low, moderate and high) and intensity of immunostaining as assessed in two subjective categories (low or strong) in comparison with the consistent immunoreactivity of positive internal controls (endothelial cells). More importantly, the clinicopathological and prognostic implications did not change for both reagents, inasmuch as the results for each antibody within individual tumours fell into the same category of immunostaining. Therefore, we indicate that both antibodies are excellent and highly reproducible reagents for immunodetecting fascin on paraffin sections, and that both are equally suitable for studying fascin expression in lung pathology.

The high prevalence of fascin immunoreactivity in NSCLC (as opposed to the lack of staining of nonneoplastic epithelia of the lower respiratory tract) and its significant relation with tumour recurrence and reduced patient survival emphasise the possible involvement of this protein in the development and progression of NSCLC. To our knowledge, this is the first study in which a large series of stage I NSCLC with long-term follow-up has been investigated for fascin immunoreactivity. Recently, Del Rosario *et al* (2001) have reported in abstract form only that fascin immunoreactivity was detectable in 31% of 55 adenocarcinomas and 89% of 55 squamous cell carcinomas, correlating significantly with high tumour grade in the former and low tumour grade in the latter. No relation was noted, however, with tumour stage or patient survival. The comparison of the data is hampered by the lack of details on the evaluation of immunostaining and on the stage distribution of the patients in the previous series. Other studies on patients with breast and ovarian cancers have failed to demonstrate any definite relation between fascin immunoreactivity and tumour grade (Grothey *et al*, 2000b; Hu *et al*, 2000). In breast cancer patients, however, fascin immunoreactivity was more prevalent in nondiploid and highly proliferating tumours with a negative hormone receptor status and Her2/neu overexpression (Sun *et al*, 1997; Grothey *et al*, 2000a,b), while in ovarian cancer it was correlated with advanced disease stage (Hu *et al*, 2000). Recently, Yamashiro *et al* (1998) have reported that fascin transfection or protein microinjection into normal epithelial or mesenchymal cell lines induced remarkable changes in cell morphology, with the emission of microspikes on apical surfaces, extended lamellipodia at the basolateral surfaces, and disruption of cell–cell contact. This supports the view that fascin overexpression may ultimately affect the differentiation (and hence the grade) of lung tumours via the remodelling of the cell shape and volume, and the changes in cell motility and aggregation status.

The prognostic power of tumour grade, growth patterns (Pastorino *et al*, 1997; Travis *et al*, 1999), and cell subtyping (Shimosato, 1994) in NSCLC is highly controversial. Fascin immunoreactivity may thus be considered a more reliable tool for better assessing the biological aggressiveness and clinical course of NSCLC. Furthermore, the inhibition of fascin activity by phosphorylation (Yamakita *et al*, 1996, Ono *et al*, 1997, Tilney *et al*, 1998; Adams *et al*, 1999, Cohan *et al*, 2001) or antisense oligonucleotide strategy (Al-Alwan *et al*, 2001) could represent potentially novel therapeutic options in the treatment of lung cancer. The validation of differentially expressed fascin levels in NSCLC could open new diagnostic and therapeutic perspectives, such as the development of radiologic imaging systems based on tagged antibody strategy to detect small lung cancers, either primary or metastatic, or the strategy of cell-mediated or antibody-based immunotherapy using fascin for targeting more aggressive lung carcinomas.

An interesting aspect of our investigation is the relation between fascin immunoreactivity and proliferative activity of tumour cells. Collectively considerated, tumours with high (>60%) fascin immunoreactivity showed significantly greater Ki-67 labelling index (mean±s.d.: 27.9±16.5%; median: 27%) than tumours with negative to low (up to 30%) fascin immunoreactivity (mean±s.d.: 20.4±13.4%; median: 20%) (*P*=0.028). The wide dispersion of values in the correlation test (*r*=0.23), however, indicated that probably there is no linear relation between the two markers, and that this association may depend on the higher tumour grade of fascin overexpressing adenocarcinomas. In fact, a closer positive relation was found between Ki-67 labelling index and tumour grade (*r*=0.44, *P*<0.001) (data not shown). As a matter of fact double immunolabelling experiments of individual tumours indicated that foci of actively proliferating tumour cells were less immunoreactive for fascin than adjacent tumour nests showing a lower proliferative activity. This could imply that cycling cells (like those collectively highlighted by the Ki-67 antigen) may transitorily downregulate fascin, with no apparent differences in immunostaining being appreciable between tumour cells clearly showing mitotic figures (corresponding to the M phase) and tumour cells in the other phases of the cell cycle (G1, G2 and S). The precise molecular mechanisms and the role of fascin expression in relation to the cell cycle of NSCLC still remain to be explained. Previous data by Duh *et al* (1994), however, indicated that fascin mRNA was highly expressed in actively growing renal carcinoma cell lines and in activated, but not in resting, lymphocytes, suggesting a functional role for this protein in proliferation. Likewise, Guvakova *et al* (2002) recently reported that the activation of insulin-like growth factor (IGF)-I receptor signalling is associated with the induction of fascin spikes and ruffles in breast cancer cell lines, without altering the protein levels. Interestingly, several lines of evidence implicate IGFs and their cognate receptors in proliferative and antiapoptotic effects of many human malignancies, including NSCLC cell lines (Lee *et al*, 2002). Therefore, we cannot exclude that other regulatory mechanisms may be involved in pulmonary adenocarcinomas, to explain our apparently conflicting findings of fascin downregulation in actively proliferating tumour cells when evaluated within individual tumours using the double immunostaining strategy. Further work is in progress in our laboratory to analyse this interesting point.

Bronchiolo-alveolar carcinomas are considered to be noninvasive tumours with lepidic spread along the alveolar septa (Travis *et al*, 1999). In our study, bronchiolo-alveolar components, with or without collapse of the underlying stroma (Noguchi *et al*, 1995), were consistently devoid of fascin immunoreactivity, contrasting with the adjacent invasive components of acinar/tubular or papillary types. The reasons of the differential distribution of fascin immunoreactivity in mucinous and nonmucinous bronchiolo-alveolar carcinomas, however, still remain elusive. Likewise, the protein was scarcely expressed in squamous cell dysplasia and *in situ* squamous cell carcinomas at different anatomical sites (cervix, larynx, lung, and skin) (data not shown), supporting the view that fascin upregulation is a late event in the sequential pathogenesis of NSCLC. The molecular mechanisms, however, leading to fascin upregulation in these tumours are presently unknown. Some studies have suggested that Her-2/Neu overexpression may increase fascin mRNA and protein levels in human breast cancer cell lines (Grothey *et al*, 2000a), but we have been unable to find any association between fascin and Her-2/Neu protein or gene status in our series of NSCLC, using both immunohistochemistry for protein overexpression and FISH analysis for gene amplification or polysomy (data not shown). Functional interactions with extracellular matrix molecules provide further mechanisms for the cellular regulation of fascin, although these adhesion conditons do not upregulate the protein but rather alter its subcellular distribution, either inducing or stabilising bundling of actin by fascin, or not supporting fascin protrusions (Kureishy *et al*, 2002). At present, evidence that fascin – a marker of tumour aggressiveness – gets turned on in NSCLC by means of the contact with extracellular matrix moieties is poor. On the contrary, some studies have recently reported that the downregulations of thrombospondin-1 (Yamaguchi *et al*, 2002) or tenascin-C (Cai *et al*, 2002), two molecules of extracellular matrix supporting the formation of large arrays of fascin microspikes and ribs at cell margins and in lamellipodia (Adams, 1995; Fischer *et al*, 1997; Adams and Schwartz, 2000; Anilkumar *et al*, 2002), are instead associated with poor prognosis and tumour recurrence in patients with NSCLC.

The correlation between fascin upregulation and metastatic potential may be at least in part justified by the peculiar prevalence of fascin expression in most neoplastic emboli inside blood vessels, independent of the tumour type, and in most endothelial cells of the pulmonary microvessels, but not of the major vessels. On the other hand, the association between fascin expression and occurrence of contralateral thoracic or distant metastases supports the view that this molecule may be implicated in metastatic spread. The metastasis mechanism could involve either direct vascular permeation by actively migrating tumour cells or development of newly formed microvessels highlighted by strong fascin immuno-reactivity. In fact, a synergism between vascular invasiveness and neoangiogenesis may actually favour metastatic spread. Further work is in progress in our laboratory to test the possibility of using fascin as a more suitable marker of neoangiogenesis than CD34 in lung cancers. Although the molecular mechanisms of fascin accumulation in tumour cell emboli of NSCLC still remain elusive, fascin overexpression by neoplastic cells, either constitutive or induced by yet unknown factors, may facilitate invasive tumour growth, vascular permeation, and ultimately the appearance of distant metastases.

In our study we did not try to compare directly the immunostaining intensity for fascin with quantitative measurements of the protein levels or the corresponding mRNA within individual tumours. As a matter of fact, in order to elucidate the prognostic role of fascin in NSCLC it was necessary to analyse retrospectively a large series of tumours with long-term follow-up (just like our series), and frozen tissue (the most suitable and reliable material for RT-PCR assessment) had not been collected at that time. Further studies, however, are in progress in our laboratory to perform mRNA fascin analysis also in archival, paraffin-embedded material. Although immunocytochemistry cannot be strictly considered a tool of quantitative analysis, we devised a simple three-tier semiquantitative scheme (negative, low, and strong) by comparing tumour cell immunostaining with internal positive controls (endothelial and dendritic cells) in order to minimise bias because of the immunostaining variability and the subjective impression of the slide readers. Our aim, however, was not to demonstrate that differences in fascin immunostaining intensity correlate with different levels of the protein (even though that is likely), but that differences in immunostaining could be reproducibly assessed to stratify the patients into different risk categories. As a matter of fact, squamous cell carcinomas – that showed the highest prevalence of fascin immunoreactivity with 86% of them being over 60% of immunolabelled cells – could be easily subclassified according to fascin immunostaining intensity into different prognostic categories. This suggests that different levels of the protein may affect the biological behaviour of squamous cell carcinoma.

In our study fascin was widely detected in most invasive squamous cell carcinomas of the lung, though with variable immunostaining intensity, whereas the protein was scarcely expressed in *in situ* squamous cell carcinomas at different anatomical sites (cervix, larynx, lung, and skin; data not shown). In experimental models, it has been demonstrated that aggregates of squamous cell carcinoma cells lose cell–cell junctions and disperse via the selective induction of fascin-rich microspikes on the cell surface because of interaction of *α*3*β*1 integrin with its ligand laminin 5 elaborated by tumour cells themselves (Kawano *et al*, 2001) and the activation of downstream signalling mechanism involving the Rho-family small GTPase Cdc42 (Machesky and Hall, 1997, Adams and Schwartz, 2000). Laminin 5 and *α*3*β*1 integrin are widely expressed in lung carcinomas, especially squamous cell carcinomas, and at the epithelial–stromal interface of tumour clusters (Bartolazzi *et al*, 1995; Maatta *et al*, 1999; Catusse *et al*, 2000). In our study, we found that fascin immunoreactivity was stronger in poorly differentiated squamous cell carcinomas and at the leading edges of infiltrating horn pearls. An increased production of laminin 5 has been reported also in other types of human squamous cell carcinomas, especially in tumours showing more infiltrative growth and poorer differentiation (Ono *et al*, 1999; Haas *et al*, 2001; Yamamoto *et al*, 2001). Therefore, we suggest that the increasing accumulation of fascin in squamous cell carcinomas of the lung may be implicated not only in the epithelial morphogenesis needed to tumour growth and stromal invasion, but also in the development of metastases when fascin is accumulated to some extent.
